# Time perception in astronauts on board the International Space Station

**DOI:** 10.1038/s41526-023-00250-x

**Published:** 2023-01-19

**Authors:** Deborah C. Navarro Morales, Olga Kuldavletova, Gaëlle Quarck, Pierre Denise, Gilles Clément

**Affiliations:** UNICAEN, INSERM, CHU Caen, Normandy University, COMETE, CYCERON, Esplanade de la Paix, 14032 Caen, France

**Keywords:** Physiology, Neuroscience, Psychology

## Abstract

We perceive the environment through an elaborate mental representation based on a constant integration of sensory inputs, knowledge, and expectations. Previous studies of astronauts on board the International Space Station have shown that the mental representation of space, such as the perception of object size, distance, and depth, is altered in orbit. Because the mental representations of space and time have some overlap in neural networks, we hypothesized that perception of time would also be affected by spaceflight. Ten astronauts were tested before, during, and after a 6–8-month spaceflight. Temporal tasks included judging when one minute had passed and how long it had been since the start of the workday, lunch, docking of a vehicle, and a spacewalk. Compared to pre-flight estimates, there is a relative overestimation for the 1-min interval during the flight and a relative underestimation of intervals of hours in duration. However, the astronauts quite accurately estimated the number of days since vehicle dockings and spacewalks. Prolonged isolation in confined areas, stress related to workload, and high-performance expectations are potential factors contributing to altered time perception of daily events. However, reduced vestibular stimulations and slower motions in weightlessness, as well as constant references to their timeline and work schedule could also account for the change in the estimation of time by the astronauts in space.

## Introduction

To construct a mental representation of our world, we perceive our environment by constantly processing sensory inputs from the visual, vestibular, and somatosensory systems. This representation is also influenced by our expectations and our experience, which are derived from our knowledge of the costs and consequences of acting in this environment. The central neurovestibular system naturally takes gravity into account during spatial orientation, balance, and motor control. This system is also indispensable for constructing our mental representation of the world. It continuously processes data from the visual, vestibular, and somatosensory channels to update our spatial maps. Previous research suggests that distances are underestimated when subjects are in weightlessness during parabolic^1,2^ or orbital^3^ flight. This distance underestimation is thought to be due to adaptive changes in the processing of gravitational information by the neurovestibular system that alter the construction of spatial maps^4,5^.

Exposure to weightlessness during spaceflight is known to elicit changes in vestibular responses, i.e., orientation illusions, errors in sensory localization, changes in vestibulo-ocular reflexes, and space motion sickness^6^. Cognitive tasks involving the neurovestibular system, such as mental rotation^7–9^, perceived orientation^10^ and judgments of distance^4^ are also affected during spaceflight. Some astronauts and cosmonauts have reported a ‘time compression syndrome’ in orbit, whereby they perceive time as compressed relative to the perceptions gained during training and simulation^11^. In weightlessness, routine tasks require different cognitive demands than they would on Earth. Astronauts in orbit also report that they require more time than normal to execute standard mental activity^12,13^ . ‘Space fog’ is another reported syndrome that affects cognitive performance during the first weeks of a mission^14^. After astronauts have adapted to weightlessness and they re-enter the atmosphere, they also encounter a condition called ‘entry motion sickness’, which slows the speed of decision making and alters their ability to control the vehicle and their movements^15^. The neurovestibular challenges that occur when the crewmember returns to normal gravity include alterations in manual control^16^, inability to egress the vehicle^17^, postural imbalance^18^, and impaired locomotion^19^.

Einstein^20^ revolutionized physics a century ago with his theory that space and time are intertwined^21^. Based on this theory, we hypothesized that the absence of gravitational reference alters the construction of the mental representation of *both* space and time. Because astronauts underestimate distances and because the pace of their motion is slower in weightlessness, we hypothesized that astronauts would also underestimate the relative time between events. Prior to the present study, subjective perception of time during long-duration spaceflight had not been investigated. The results of this study have operational implications: altered perception of time during spaceflight might impact operations that require critical timing, such as docking operations and piloted landings.

## Methods

### Participants

10 healthy crewmembers (9 male, 1 female; age *M* = 44.1, SD = 4.6) who flew on the International Space Station (ISS) participated in this study. All crewmembers passed a United States Air Force Class III medical examination and had no known history of vestibular or oculomotor abnormalities. 15 healthy subjects (6 females, 9 males; age *M* = 43.2, SD = 18.8) participated in a control study in the laboratory.

The test procedures were approved by the European Space Agency Medical Board and the NASA Johnson Space Center Institutional Review Board and were performed in accordance with the ethical standards laid down in the 1964 Declaration of Helsinki. All subjects gave a written informed consent before participating in the study. Informed consent was obtained from the subject for publication of identifying information/images in an online open-access publication.

### Experimental protocol

In the flight study, the tests were performed before, during, and after 6–8-month spaceflights (*M* = 202, SD = 28 days). The pre-flight test sessions occurred at launch minus (L-) 205 ± 51 days, L-149 ± 55 days, and L-116 ± 45 days. In-flight test sessions were conducted approximately every month: i.e., on flight day (FD) FD17 ± 6 (M ± SD), FD46 ± 8, FD71 ± 6, FD99 ± 7, FD134 ± 8, and FD164 ± 7. After the astronauts returned to Earth, tests were performed at return plus (R + ) 1 day, R + 5 ± 1 day, and R + 9 ± 1 day.

A psychophysics test was administered, during which the subjects were asked to judge when one minute had passed. During this test, subjects wore a head-mounted display (Oculus Rift, Oculus VR, Menlo Park, CA), and used a finger trackball connected to a laptop to report their responses. Subjects were wearing earphones for listening to the instructions and for attenuating/masking noises from the spacecraft. Using the finger mouse, they pressed a ‘go’ button and waited one minute before pressing on a ‘stop’ button. Only the ‘go’ and’stop’ buttons were displayed during the test. Subjects were not allowed to count the seconds passing by.

**Fig. 1:**
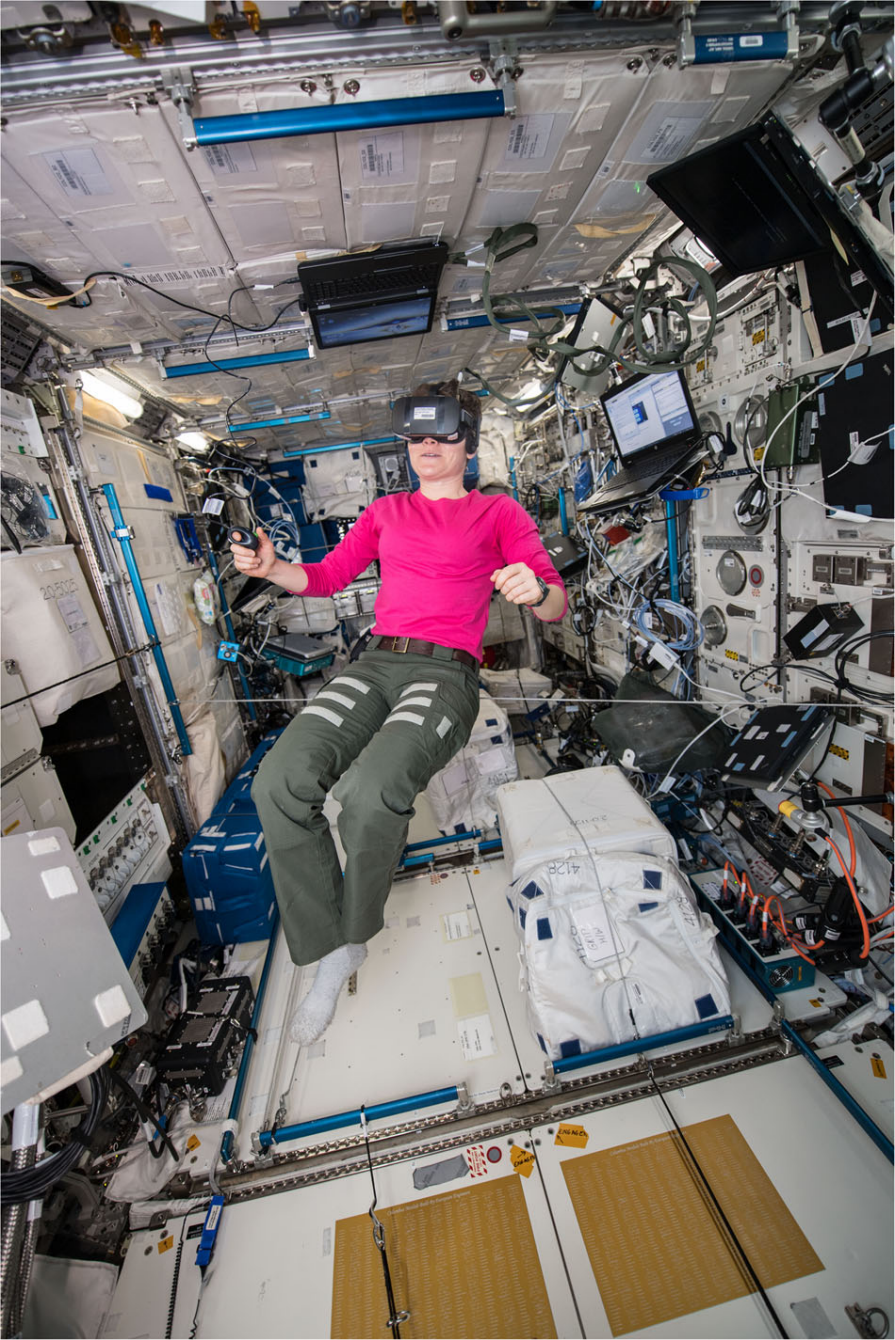
Astronaut performing the experiment while free-floating on board the ISS. Photo credit NASA.

On the ground, this test was performed in the seated upright position; on the ISS, astronauts were in the free-floating conditions (Fig. 1). During the free-floating conditions there are no proprioceptive, tactile, or static vestibular cues that participate in spatial orientation. Previous studies have demonstrated that the perception of distance and the depth of objects are altered when free-floating in orbit^3,4^. To investigate the relationship between these changes in spatial perception and changes in time perception, the psychophysics test was performed in the same conditions as the previous spatial perception tests.

The second part of the experiment included a series of questions to document the potential changes in the astronauts’ perception of longer periods of time, i.e. hours and days, while in orbit. The subjects doffed the head-mounted display and used the laptop keyboard to answer the following 5 questions: (a) How long has it been since the last time you performed this test?; (b) How long has it been since you started your work day?; (c) How long has it been since you had your lunch?; (d) How long has it been since the last vehicle docked at the ISS?; and (e) How long has it been since the last extra-vehicular activity (EVA)?

In the flight study, the start of the workday was defined as the termination of the morning daily planning conference (DPC), which occurred about one hour after the crew awoke. The time of lunch was determined by the astronauts’ daily schedule. The dates of docking and EVA was determined from the Flight Program Integration Panel document provided by NASA Mission Integrations and Operations Office. Because this information was not available before and after the flight, the questionnaire was only administered during the flight.

In the ground-based study, the subjects performed the ‘How Long is a Minute?’ test in the laboratory while sitting upright and using identical computer hardware and software as on board the ISS. The subjects were also asked the perceived number of days since the last test session and how many minutes since they woke up and had breakfast (which they noted in a diary). The number of days between test sessions of the control subjects in the laboratory (*M* = 44.1, SD = 10.2) was similar to the number of days separating the 3 pre-flight sessions with the astronauts (*M* = 45.2, SD = 28.4), and so were the durations since wake up and lunch.

### Statistical analysis

The errors between the perceived durations and the actual durations were calculated and time errors were computed in terms of percentage or days.

First, linear mixed models (LMM) were used to compare the ground-based responses of the 2 subject groups (astronauts and controls) and to establish whether they differed, and whether the results of the 3 test sessions differed (dependent variable: time error; fixed effects: tests sessions; group: astronauts or controls; random effects: subjects). A second set of LMM was used to compare measurements from different sessions within the flight phases (pre, in, post) in astronauts (dependent variable: time error; fixed effect: test sessions; random effects: subjects). A third set of LMM was used to compare the time errors during the pre-, in-, and post-flight sessions (depended variable: time error; fixed effects: flight phase; random effects: subjects). *Post-hoc* pairwise comparisons were then conducted using Bonferroni adjustment for multiple comparisons. Fixed effects estimates, confidence limits, and random effects standard deviations of these LMMs are provided in Supplementary Table 1.

When preflight measurements were not available in the astronauts (perceived durations since beginning of workday and lunch), an independent Sample Mann–Whitney test was used to compare their inflight responses with those of the control subjects on the ground. When these responses were not available in the control subjects neither (perceived durations since last docking and last EVA) a one-sample t-test was used to determine if the time errors during the flight were different from zero. Statistical analysis was performed with JASP 0.16.1.0 and IBM SPSS Statistics 27.0 software.

### Reporting summary

Further information on research design is available in the Nature Research Reporting Summary linked to this article.

## Results

### How long is a minute?

**Fig. 2:**
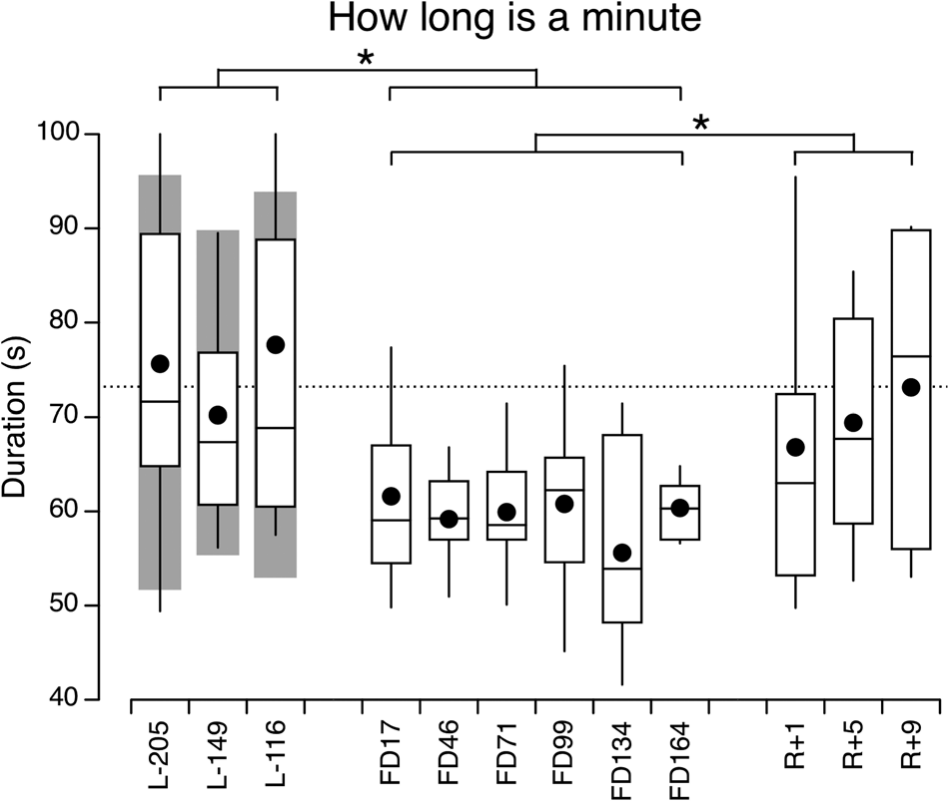
How long is a minute. Box and whisker plots of 10 astronauts’ perceived duration of one minute before (L−), during (FD), and after (R+) spaceflight. Filled symbol represents the mean, center line represents the median, bounds of box represent the first and third quartiles, and whiskers represent the minimum and maximum values in the set. The dotted line represents the average of all pre-flight measurements in the astronaut group. The gray area represents the mean ± standard deviation of measurements taken from 15 control subjects in the laboratory during 3 test sessions separated by approximately one month. L− Days before launch, FD Flight days, R+ Days after return. *p < 0.05 (linear mixed model).

On the ground, on average subjects indicated the duration of one minute to be 74.1 ± 19.5 s (mean ± SD) (Fig. 2). The LMM indicated no significant differences between the 3 test sessions in the control subjects and the astronauts [*F*(2,46) = 0.58, *p* = 0.56], which suggests that there was no learning or adaptive effect due to the repetition of the test. No significant differences were detected between the 2 groups of subjects [*F*(1,23) = 0.008, *p* = 0.928].

A LMM on the perceived durations of 1 min during the test sessions performed within each flight phase indicated that there were no significant differences within the sessions pre-flight [*F*(2,18) = 0.939, *p* = 0.409], in-flight [*F*(5,45) = 0.593, *p* = 0.705] and post-flight [*F*(2,18) = 1.212, *p* = 0.321]. When measurements within each flight phases were pooled, the LMM indicated a significant main effect of flight phases [*F*(2,108) = 15.050, *p* < 0.001]. *Post-hoc* tests indicated that this difference was significant between pre-flight and in-flight (*p* < 0.001) and between post-flight and in-flight (*p* = 0.002), but not significant between pre-flight and post-flight (*p* = 0.484). During the flight, the averaged perceived duration of one minute was 59.6 ± 9.1 s (mean ± SD), which corresponds to a 20.0% decrease from before flight (74.5 ± 20.2 s). Interestingly, although the perceived duration of one minute was less during flight relative to before flight, the perception during flight was essentially accurate throughout the 6 in-flight sessions.

### Duration between test sessions

**Fig. 3:**
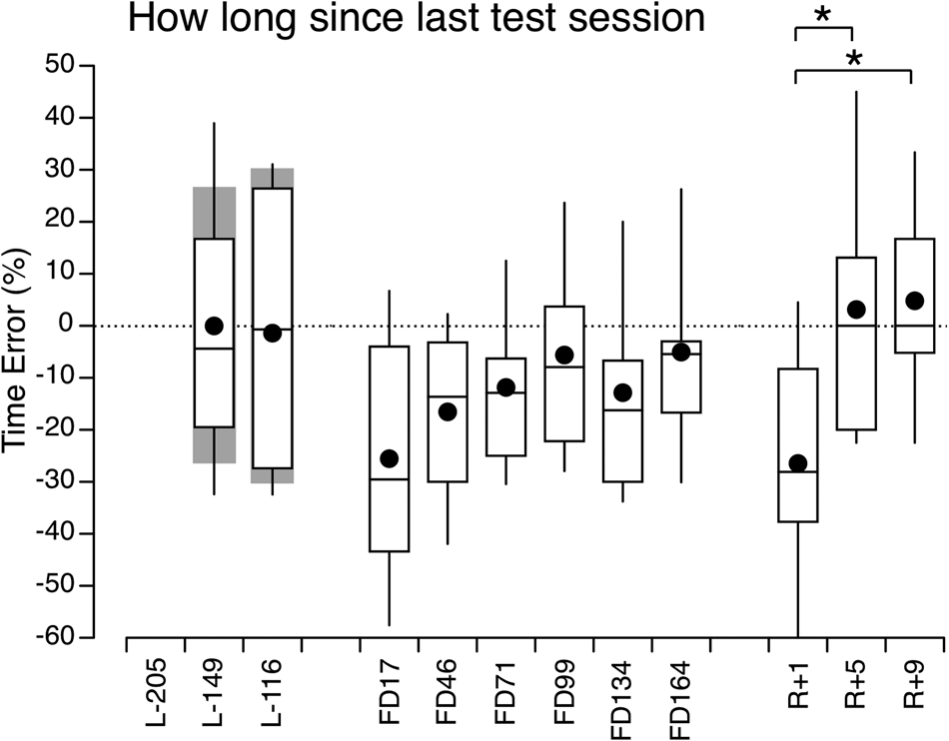
How long since last test session. Box and whisker plots of the error in 10 astronauts’ perceived duration since the last test session before (L−), during (FD), and after (R+) spaceflight. Filled symbol represents the mean, center line represents the median, bounds of box represent the first and third quartiles, and whiskers represent the minimum and maximum values in the set. The dotted line represents the average of all pre-flight measurements in the astronaut group. The gray area represents the mean ± standard deviation of measurements taken from 15 control subjects in the laboratory. *p < 0.05 (linear mixed model).

The time interval between the test sessions was approximately the same before flight (34–56 days) and for sessions FD45 to R + 1 (31.0 ± 10 d). The time interval between the last pre-flight session (L-116) and FD17 was much longer (133.8 ± 43 d). When comparing the astronaut’s perceived duration between the test sessions, the largest time errors occurred on FD17 and R + 1, i.e., shortly after the transition in gravity levels (Fig. 3). The mean time error for these two sessions was −26.0% (SD = 24.3).

The LMM indicated no significant differences between the 2 test sessions in the control subjects and the astronauts [*F*(1,23) = 0.023, *p* = 0.881], which suggests that there was no learning or adaptive effect due to the repetition of the test. No significant differences were detected between the 2 groups of subjects [*F*(1,23) = 0.005, *p* = 0.942].

We compared whether the perceived durations were different within each flight phase. A LMM indicated that these differences were only significant within the post-flight measurements [*F*(2,27) = 4.913, *p* = 0.009), and particularly between R + 1 and R + 4 (*p* = 0.018) and between R + 1 and R + 8 (*p* = 0.027). We then pooled measurements within each flight phases and we conducted another LMM, which indicated no significant main effect of flight phases [*F*(2,98) = 2.390, *p* = 0.097].

### Duration since start of workday and lunch

**Fig. 4:**
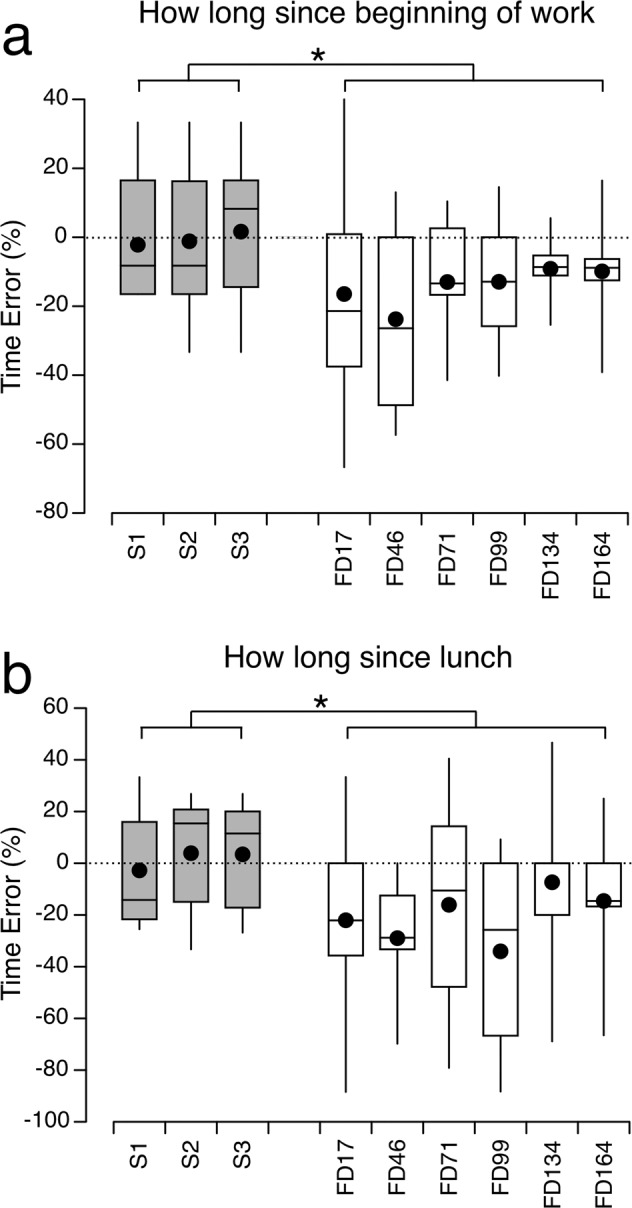
Time error for durations in hours. Box and whisker plots of the error in perceived duration since the start of the work day (**a**) and since lunch (**b**) in 10 astronauts during spaceflight and 15 subjects during 3 sessions (S1, S2, S3) in the laboratory. Filled symbol represents the mean, center line represents the median, bounds of box represent the first and third quartiles, and whiskers represent the minimum and maximum values in the set. *p < 0.05 (Sample Mann–Whitney test).

On the ISS, a typical workday begins with the morning DPC between the astronauts and the mission control center. On average, this experiment took place 4.4 ± 1.0 h (mean ± SD) after the end of the DPC. The same interval was used when testing the control subjects in the laboratory (4.5 ± 1.1 h). Two separated LMMs indicated that there were no significant differences in the perceived duration since the beginning of the workday between the 3 pre-flight sessions in the control subjects [*F*(2,42) = 0.286, *p* = 0.880], and between the 6 pre-flight sessions in the astronauts [*F*(5,54) = 0.469, *p* = 0.797). However, there were significant differences between the perceived durations of the astronauts in-flight and the control subjects on the ground (Mann–Whitney, *p* = 0.001) (Fig. 4a). Overall, the astronauts underestimated the duration since the beginning of their workday by −14.2% (SD = 24.2%).

On average, the experiment occurred 2.7 ± 0.7 h (mean ± SD) after lunch in orbit and 2.5 ± 0.5 h after lunch in the laboratory. Two separated LMMs indicated that there were no significant differences between the 3 pre-flight sessions in the control subjects [*F*(2,42) = 0.014, *p* = 0.986], and between the 6 in-flight sessions in the astronauts [*F*(5,45) = 0.591, *p* = 0.707]. However, there were significant differences between the perceived durations of the astronauts in-flight and the control subjects on the ground (Mann–Whitney, *p* = 0.037) (Fig. 4a, b). Overall, the astronauts underestimated the duration since their lunch by −19.2% (SD = 36.1%).

It is possible, however, that the astronauts could have underestimated the duration after the start of their workday and/or lunch before the flight. Unfortunately, we could not measure these durations because we did not have access to the astronaut’s schedule during their training.

### Duration since last docking and extra-vehicular activity (EVA)

**Fig. 5:**
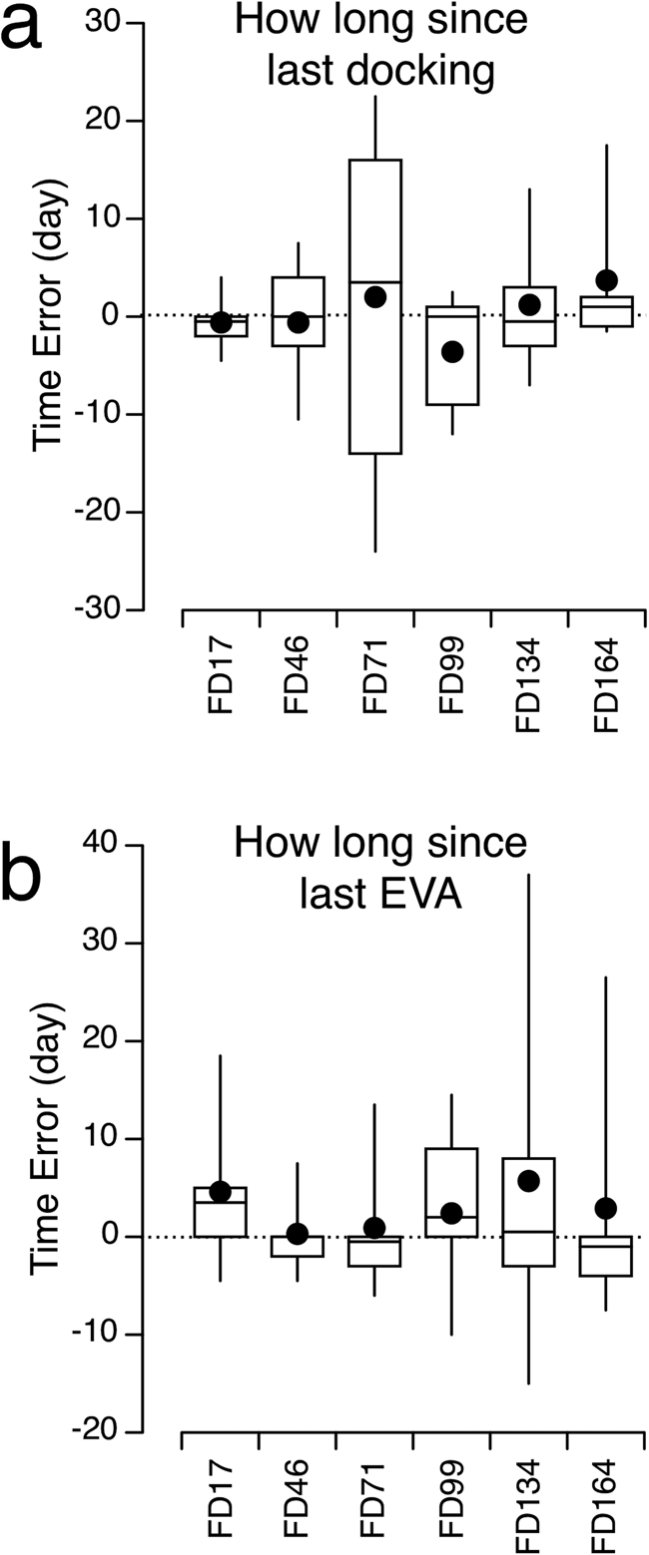
Time error for durations in days. Box and whisker plots of the error in 10 astronauts’ perceived duration since the last vehicle docking to the ISS (**a**) and the last spacewalk (Extra-Vehicular Activity, EVA) (**b**). Filled symbol represents the mean, center line represents the median, bounds of box represent the first and third quartiles, and whiskers represent the minimum and maximum values in the set. *p < 0.05 (one-sample t-test).

The docking of a vehicle to the ISS occurred an average of 26.0 ± 12.4 days (mean ± SD) before administering the questionnaire. Astronauts were quite accurate in their estimations of this duration: their errors were less than one day (+2.2%) (Fig. 5a). A LMM indicated that there were no significant differences in the perceived duration since the last docking between the 6 in-flight sessions [*F*(5,45) = 0.695, *p* = 0.630]. However, this time error was not significantly different from zero (t-test, *p* = 0.797).

Similarly, the EVAs occurred an average of 49.4 ± 15.9 days (mean ± SD) before administering the questionnaire. The time error for the estimations of this duration was 2.8 ± 11.9 days (+5.6%) (Fig. 5b). A LMM indicated that there were no significant differences in the perceived duration since the last EVA between the 6 in-flight sessions [*F*(5,45) = 0.312, *p* = 0.903]. However, this time error was not significantly different from zero (t-test, *p* = 0.167).

## Discussion

This study indicates that astronauts’ perceived the duration of one minute to be less during spaceflight (−20.0%) than before flight. In addition, they underestimated durations ranging from 2 h (since lunch) and 5 h (since start of their workday) by −36.1% and −24.2%, respectively. They also underestimated the time elapsed since the last test session when there was a change in gravity level between these sessions (−26.5% on R + 1). However, they were essentially correct in estimating the durations in days elapsed since the last docking of a vehicle to the ISS (+2.2%) and since the last EVA (+5.6%).

The method for assessing time perception of a 1-min time period is the method of production, i.e. indicating a 1-min interval by pressing a button. Before the flight, a clock time of on average 74.1 s was judged as 60 s on average by the subjects. In other words, at a clock time of 60 s the subjects still thought it was perhaps 50 s, and waited longer before pressing the button. Similarly, the relative underproduction during the flight by the astronauts (59.6 s) as compared to before (74.5 s) refers to a relative overestimation of duration. In other words, astronauts in space feel that more time (the 60 s) has gone by after 60 s (they then pressed the button) than the astronauts on the ground who at 60 s felt a relatively shorter duration (say, 50 s) and waiting a little longer to press the button (on average at 74.5 s). This is a classic dissociation in the interpretation of a time production and a time estimation task. In the latter the observer waits through the designated time and then verbally reports clock time (‘about 60 s).

The fact that there is a relative overestimation for the 1-min interval is not at odds with the relative underestimation of intervals in the hours range. There is ample evidence that different time intervals are governed by different mechanisms. The 1-min interval could still be just within the working memory span (and a clock mechanism could apply) but the hour time range is way beyond and memory processes apply^22,23^.

Why are the astronauts actually more accurate in their estimate of minutes during spaceflight? One possible interpretation is related to the way the astronauts’ work is organized on board the ISS. By every ISS workstation, there is a daily minute-by-minute schedule displayed on a computer (the Onboard Short-Term Plan Viewer, or OSTPV). Superimposed on this schedule is a vertical red bar that moves from left to right and symbolizes the passing of time. Each day, the astronauts perform different activities (e.g. experiments, equipment maintenance, taking photographs, etc.) scheduled at a given time for a given duration. Consequently, crewmembers may have lunch at different time from one day to another. The large variability in the astronauts’ time errors for estimating the time elapsed since the beginning of the workday and lunch is presumably due to the different type and schedule of activities they perform each day. The red bar displayed on the OSTPV indicates when crewmembers must start and end each activity. The speed of the red bar movement, and the comparison between the perceived time for completing an activity and the actual time when crewmember looks at the OSTPV presumably contribute to them becoming more accurate in their estimates of minutes during spaceflight.

According to Einstein’s theory of relativity^20^, an observer traveling at high speed will experience time passing more slowly than an observer at rest. At the speed of the International Space Station (28,160 km/h), this difference is in the order to 4 ×10^−11^ s. This effect is negligible in our results, which show changes in the order of minutes or days.

A confounding factor for accurate time perception during space operations is the fact that different time scales are used on the ISS, which can be confusing. The official time is Greenwich Mean Time (GMT), but the astronauts and cosmonauts often use Houston time (GMT-5 h) or Moscow time (GMT + 2 h) to communicate with mission control centers and their families. Also, critical operations such as vehicle docking and EVA are documented in mission elapsed time, i.e. the time elapsed since the launch or entry into the airlock.

The conditions during spaceflight, including weightlessness, prolonged isolation in confined areas, stress related to workload, and high-performance expectations, are known to affect human physiological and psychological responses^6,12^. These conditions could also alter the perceived temporal relationships between events. During his 8-min EVA, Alexey Leonov^24^ clearly experienced an underestimation of time: ‘I was disappointed,’ he wrote in his post-flight report, ‘that the time assigned for working outside the craft flew by very quickly. The entire period I remained in outer space seemed to be only 1 or 2 min.’

Only 2 studies have been performed previously to assess time perception during spaceflight. The first experiment took place after the historical one-orbit flight of Yuri Gagarin, when Gherman Titov flew on the Vostok-2 for a full day (17 Earth orbits). The objective was to assess his ability to evaluate time intervals. After starting a stopwatch, he began to count 20 s in his head; when he estimated subjectively that 20 s had passed, he stopped the stopwatch and looked at the actual elapsed time. The results were recorded in his onboard diary. The average time estimates during the 4 in-flight sessions were 20.3, 20.2, 20.1, and 20.1 s. These estimates were not significantly different from those measured during training, but they were biased by the fact that he was counting in his head and he had continuous feedbacks on his performance^24^.

The second experiment on time perception during spaceflight was performed on 4 astronauts during a 4-day Space Shuttle mission. The test was a classic time reproduction (non-counting) task. Subjects viewed a visual target traversing a display and, while it was obscured, estimated the time of its arrival at a predetermined point by any means other than counting. The target moved at various speeds, so that the duration of the task ranged from 2 to 16 s. The 4 astronauts were tested the day before their flight, each day during the flight, 3 h after landing, and again 3 days later^25^. As the time duration of the task increased, the subjects tended to underestimate duration and these errors in duration estimates increased each day as the flight progressed. Three hours after landing the duration estimates were also significantly larger than on FD4^25^.

The results of the above experiment and the present study suggest that the ability to estimate brief intervals of time deteriorates during a space mission and shortly after landing. Similar effects were also observed in subjects exposed to hypergravity in a centrifuge^11^. A potential consequence of these effects is that crewmembers who need to make quick decisions and perform critical tasks during flight and re-entry may exhibit some delays in their responses, which could compromise safety.

Gibson^26^ pointed out that some measures of time are intrinsic, i.e., they are physical phenomena ‘out there’ in the world. These include the year (the Earth’s rotation around the sun which, due to the tilt of the Earth’s axis, yields a sequence of seasons), the month (the Moon’s rotation around the Earth, with its visible phases), and the day (the Earth’s rotation around its own axis, yielding dawn, Noon, dusk, and so on). These intrinsic measures of time contrast with the extrinsic measures of time, such as seconds, minutes, and hours. Whereas the duration of the year, the month, and the day are fixed by physical facts, the duration of the second is arbitrary; it is a convention that works solely because many people have agreed to it. The duration of the second can be changed. We could, if we wished, have only 10 h in the day, with minutes and seconds that were much longer. By contrast, we cannot change the physical duration of the day. The day is an event, in Gibson’s sense, and is perceivable as any physical event. The second is not an event—it does not exist ‘out there’ to be perceived, but exists only in the mind, as a social convention. In addition, circadian and circalunar rhythms follow 24-h and 30-day cycles, respectively. The results of our study indicate that astronauts are quite accurate when estimating intrinsic measures of time (days, months), but are inaccurate when estimating extrinsic measures of time (seconds, minutes, hours). However, the accuracy when estimating intrinsic measures of time (days) is altered following transitions between gravity levels (0 g to 1 g).

The results of psychophysical experiments on Earth indicate that subjects have distorted time perception when they are in stressful situations^27^. For example, during dangerous events such as plane and car accidents, many people report an overestimation to time: fractions of seconds can be perceived as minutes^28^. During parachute jumps, subjects tend to overestimate the time intervals and delay the opening of their parachute^24^. By contrast, the results of our study indicate that subjects either underestimate periods of time, or report accurate time perception. It is therefore unlikely that the results of this study are due to the stress of spaceflight.

Depending on the situation, time is perceived as either passing slowly or flying by. When we are bored, we feel that time passes more slowly than when we are entertained. Impulsive people feel that time is excruciatingly slow when nothing is happening^22^. Time experience and time judgments are also altered in depressive and manic patients^29^. In general, institutionalized individuals, such as individuals in homes for the elderly, whose days are highly regulated and monotonous, experience time as passing slowly, i.e. they overestimate time durations^30,31^. But the same individuals may also experience time as speeding up when enjoyable and memorable events occur, such as a visit from family members or social events^32,33^. This effect is also likely to happen during spaceflight. As indicated above, astronauts’ working days are filled with various activities in a timeline that changes from one day to another. During this busy schedule, time is perceived as going faster, and durations in minutes or hours are therefore underestimated^34^. Whereas, when unique events take place, such as vehicle docking and EVA, these days are memorized more accurately.

Vicario et al.^35^. showed that optokinetic stimulation influences time perception. Subjects overestimated time intervals after optokinetic stimulation compared with their estimations before optokinetic stimulation. Because optokinetic stimulation interacts with the vestibular system, for example by generating nystagmus or illusion of self-motion (vection), this result suggests that a stimulation of the vestibular system affects the perception of time intervals. In agreement with this hypothesis, Binetti et al.^36^. found that subjects overestimated time duration during vestibular stimulation produced by whole-body rotations.

The role of the vestibular system in cognitive processes such as spatial orientation, navigation, object recognition, and memory, has been demonstrated in many studies^37^. For example, patients with central vestibular lesions exhibit difficulties with counting tasks, immediate and short-term memory^38–40^, and alterations in the perception of size and distance of objects^41^. The central neurovestibular system naturally takes gravity into account for spatial orientation, balance, and motor control. This system continuously processes data from the visual, vestibular, and somatosensory channels to update our spatial maps. Previous research suggests that astronauts underestimate distances in weightlessness^3,4^. The interpretation for this distance underestimation is that the adaptive changes in the processing of gravitational information by the neurovestibular system alter the construction of spatial maps. One plausible interpretation for the results of the present study is that the absence of gravitational reference alters the construction of the mental representation of temporal events, and this results in underestimation of the relative time between events. Due to the absence of static otolith inputs at rest, the vestibular system is less stimulated in weightlessness than in normal gravity. In addition, motions are slower in weightlessness: it takes about 50% more time in orbit to execute the same experimental procedures as on Earth. Less stimulation of the vestibular system could lead to an underestimation of the duration of time in weightlessness. In agreement with this hypothesis, perception of both distances^1,2^ and durations^42^ are altered during transient exposure to weightlessness in parabolic flight.

Our previous studies indicated that astronauts’ subjective perception of their body motion and position and the size and distance of objects were altered during spaceflight^3,4^. The present study shows that astronauts’ perception of durations ranging from one minute to several hours (which are human conventions) is also altered during long-duration spaceflight, and strikingly so in about the same percentage as for perceived distances^3^. No significant differences were seen across flight days, which indicate that these alterations occurred within 2 weeks in orbit and that no adaptation took place during long-duration spaceflight. This observation raises operational concerns regarding the ability for crewmembers to manually perform docking and landing maneuvers after two weeks in orbit without any assistance.

These results also support the existence of an overlapping perception of time and space. It has been proposed that representations of space and time both share the same metrics and cortical network, presumably located in the right parietal cortex (for stimuli shorter than one-second duration)^43^. A potential neuronal basis for the interaction between these representations comes from neurophysiological recordings in rodents hippocampus and enthorhinal cortex (which have connections with the parietal cortex) showing that place and grid cells can simultaneously code for space and time^44,45^. In agreement with this interpretation, recent brain imaging studies have shown that the spontaneous or evoked activity in the right parietal cortex was modified in astronauts after spaceflight^46–50^.

## Supplementary information

Results of the linear mixed models

Reporting Summary

## Data Availability

Data will be made available on reasonable request to Dr. Gilles Clement (gilles.clement@unicaen.fr).
