# Supplementary material for: Time perception in astronauts on board the International Space Station
Source: NPJ Microgravity. 2023 Jan 19;9:6. doi: 10.1038/s41526-023-00250-x (PMC9852442; doi:10.1038/s41526-023-00250-x)
Supplement: Supplementary file 1 — Results of the linear mixed models [file 41526_2023_250_MOESM1_ESM.pdf]

SUPPLEMENTARY TABLE 1. Fixed effects estimates, random effects standard deviations (SD), 95% confidence intervals (CI), degrees of freedom (df), t-values and p-values of the linear mixed models used. * p < 0.05, ** p < 0.01, *** p < 0.001.

**Fixed Effects**	**Estimate**	**SD**	**95% CI Lower Limit**	**95% CI Upper Limit**	**df**	**t**	***p***	
1. Effects of tests sessions and/or group on the error in perceived duration preflight (%)								
1.1. How long is a minute								
Intercept (controls; L-205)	74.340	5.173	64.009	84.671	65.132	14.371	0.000	***
Astronauts	1.816	7.725	-13.635	17.267	60.117	0.235	0.815	
L-149	-0.687	6.841	-14.386	13.012	56.876	-0.100	0.920	
L-116	-0.813	6.841	-14.512	12.886	56.876	-0.119	0.906	
L-116 * Astronauts	2.839	10.816	-18.821	24.500	56.876	0.263	0.794	
L-149 * Astronauts	-4.731	10.816	-26.392	16.929	56.876	-0.437	0.663	
1.2. Duration between test sessions								
Intercept (controls; L-149)	0.380	7.226	-14.175	14.935	44.902	0.053	0.958	
Astronauts	-0.586	10.919	-22.711	21.540	36.952	-0.054	0.958	
L-116	-1.013	9.676	-20.685	18.658	33.675	-0.105	0.917	
1.3. Start of the workday (control subjects only)								
Intercept (L-205)	-2.193	5.541	-13.376	8.989	42	-0.396	0.694	
L-149	1.027	7.837	-14.788	16.842	42	0.131	0.896	
L-116	3.827	7.837	-11.988	19.642	42	0.488	0.628	

1.4. Duration since lunch (control subjects only)

Intercept (L-205)	-2.193	5.541	-13.376	8.989	42	-0.396	0.694	
L-149	1.027	7.837	-14.788	16.842	42	0.131	0.896	
L-116	3.827	7.837	-11.988	19.642	42	0.488	0.628	

---

2. Effects of tests sessions within flight phases on the error in perceived duration (%)

2.1. How long is a minute

2.1.1. Preflight

Intercept (L-205)	75.611	6.529	61.698	89.524	15.051	11.580	0.000	***
L-149	-5.418	5.617	-17.218	6.382	18.000	-0.965	0.347	
L-116	2.026	5.617	-9.774	13.826	18.000	0.361	0.723	

2.2.2. Inflight

Intercept (FD17)	61.598	2.938	55.699	67.497	50.505	20.967	0.000	***
FD46	-2.415	3.903	-10.275	5.445	45.000	-0.619	0.539	
FD71	-1.673	3.903	-9.533	6.187	45.000	-0.429	0.670	
FD99	-0.701	3.903	-8.561	7.159	45.000	-0.180	0.858	
FD134	-6.005	3.903	-13.865	1.855	45.000	-1.539	0.131	
FD164	-1.233	3.903	-9.093	6.627	45.000	-0.316	0.754	

2.1.3. Postflight

Intercept (R+1)	66.793	4.928	56.257	77.329	14.483	13.555	0.000	***
R+5	2.608	4.079	-5.962	11.178	18.000	0.639	0.531	
R+9	6.318	4.079	-2.252	14.888	18.000	1.549	0.139	

2.2. Duration between test sessions

2.2.1. Preflight								
Intercept (controls; L-149)	-0.030	8.351	-17.582	17.522	17.914	-0.004	0.997	
L-116	-1.400	11.394	-27.174	24.374	9.000	-0.123	0.905	
2.2.2. Inflight								
Intercept (FD17)	-25.560	6.458	-38.509	-12.611	53.832	-3.958	0.000	***
FD46	9.007	9.017	-9.153	27.167	45.000	0.999	0.323	
FD71	13.705	9.017	-4.455	31.865	45.000	1.520	0.136	
FD99	19.928	9.017	1.768	38.088	45.000	2.210	0.032	*
FD134	12.685	9.017	-5.475	30.845	45.000	1.407	0.166	
FD164	20.497	9.017	2.337	38.657	45.000	2.273	0.028	*
2.2.3. Postflight								
Intercept (R+1)	-26.479	7.424	-41.712	-11.246	27	-3.567	0.001	**
R+5	29.605	10.499	8.063	51.147	27	2.820	0.009	**
R+9	31.271	10.499	9.729	52.813	27	2.978	0.006	**
2.3. Start of the workday (inflight only)								
Intercept (FD17)	-16.454	7.839	-32.170	-0.738	54	-2.099	0.041	
FD46	-7.323	11.086	-29.548	14.902	54	-0.661	0.512	
FD71	3.478	11.086	-18.747	25.703	54	0.314	0.755	
FD99	3.537	11.086	-18.688	25.762	54	0.319	0.751	
FD134	7.315	11.086	-14.910	29.540	54	0.660	0.512	
FD164	6.567	11.086	-15.658	28.792	54	0.592	0.556	
2.4. Duration since lunch (inflight only)								
Intercept (FD17)	-22.046	11.695	-45.613	1.521	44.228	-1.885	0.066	

FD46	-6.914	14.699	-36.519	22.691	45.000	-0.470	0.640	
FD71	5.965	14.699	-23.640	35.570	45.000	0.406	0.687	
FD99	-3.843	14.699	-33.448	25.762	45.000	-0.261	0.795	
FD134	14.687	14.699	-14.918	44.292	45.000	0.999	0.323	
FD164	7.445	14.699	-22.160	37.050	45.000	0.507	0.615	
2.5. Duration since last docking (inflight only)								
Intercept (FD17)	-0.600	3.072	-6.758	5.558	53.887	-0.195	0.846	
FD46	0.001	4.298	-8.657	8.657	45.000	0.000	1.000	
FD71	2.600	4.298	-6.057	11.257	45.000	0.605	0.548	
FD99	-3.000	4.298	-11.657	5.657	45.000	-0.698	0.489	
FD134	1.800	4.298	-6.857	10.457	45.000	0.419	0.677	
FD164	4.300	4.298	-4.357	12.957	45.000	1.000	0.322	
2.6. Duration since last EVA (inflight only)								
Intercept (FD17)	0.900	3.879	-6.883	8.683	52.454	0.232	0.817	
FD46	3.700	5.271	-6.917	14.317	45.000	0.702	0.486	
FD71	-0.600	5.271	-11.217	10.017	45.000	-0.114	0.910	
FD99	1.500	5.271	-9.117	12.117	45.000	0.285	0.777	
FD134	4.800	5.271	-5.817	15.417	45.000	0.911	0.367	
FD164	2.000	5.271	-8.617	12.617	45.000	0.379	0.706	

3. Effects between flight phases on the error in perceived duration (%)

3.1. How long is a minute								
Intercept (preflight)	74.480	3.024	68.294	80.667	28.914	24.626	0.000	***
Inflight	-14.887	2.894	-20.624	-9.149	108.000	-5.143	0.000	***
Postflight	-4.712	3.342	-11.337	1.913	108.000	-1.410	0.161	

3.2. Duration between test sessions								
Intercept (preflight)	-0.730	5.379	-11.439	9.979	78.201	-0.136	0.892	
Inflight	-12.193	5.937	-23.975	-0.411	98	-2.054	0.043	*
Postflight	-5.457	6.638	-18.629	7.715	98	-0.822	0.413	
